# Associations between genetic variants in mRNA splicing-related genes and risk of lung cancer: a pathway-based analysis from published GWASs

**DOI:** 10.1038/srep44634

**Published:** 2017-03-17

**Authors:** Yongchu Pan, Hongliang Liu, Yanru Wang, Xiaozheng Kang, Zhensheng Liu, Kouros Owzar, Younghun Han, Li Su, Yongyue Wei, Rayjean J. Hung, Yonathan Brhane, John McLaughlin, Paul Brennan, Heike Bickeböller, Albert Rosenberger, Richard S. Houlston, Neil Caporaso, Maria Teresa Landi, Joachim Heinrich, Angela Risch, Xifeng Wu, Yuanqing Ye, David C. Christiani, Christopher I. Amos, Qingyi Wei

**Affiliations:** 1 Duke Cancer Institute, Duke University Medical Center, Durham, NC, USA; 2 Affiliated Hospital of Stomatology, Nanjing Medical University, Nanjing, China; 3 Department of Medicine, Duke University School of Medicine, Durham, NC, USA; 4 Department of Biostatistics and Bioinformatics, Duke University Medical Center, Durham, NC, USA; 5 Department of Biomedical Data Science, Geisel School of Medicine, Dartmouth College, Hanover, USA; 6 Massachusetts General Hospital, Boston, Massachusetts, USA; 7 Department of Environmental Health, Harvard School of Public Health, Boston, Massachusetts, USA; 8 Lunenfeld-Tanenbaum Research Institute of Mount Sinai Hospital, Toronto, Ontario, Canada; 9 Public Health Ontario, Toronto, Ontario, Canada; 10 Genetic Epidemiology Group, International Agency for Research on Cancer (IARC), Lyon, France; 11 Department of Genetic Epidemiology, University Medical Center, Georg-August-University Göttingen, Göttingen, Germany; 12 Division of Genetics and Epidemiology, the Institute of Cancer Research, London, United Kingdom; 13 Division of Cancer Epidemiology and Genetics, National Cancer Institute, National Institutes of Health, Bethesda, MD, USA; 14 Helmholtz Centre Munich, German Research Centre for Environmental Health, Institute of Epidemiology I, Neuherberg, Germany; 15 Department of Molecular Biology, University of Salzburg, Salzburg, Austria; 16 Department of Epidemiology, University of Texas MD Anderson Cancer Center, Houston, Texas, USA

## Abstract

mRNA splicing is an important mechanism to regulate mRNA expression. Abnormal regulation of this process may lead to lung cancer. Here, we investigated the associations of 11,966 single-nucleotide polymorphisms (SNPs) in 206 mRNA splicing-related genes with lung cancer risk by using the summary data from six published genome-wide association studies (GWASs) of Transdisciplinary Research in Cancer of the Lung (TRICL) (12,160 cases and 16,838 controls) and another two lung cancer GWASs of Harvard University (984 cases and 970 controls) and deCODE (1,319 cases and 26,380 controls). We found that a total of 12 significant SNPs with false discovery rate (FDR) ≤0.05 were mapped to one novel gene *PRPF6* and two previously reported genes (*DHX16* and *LSM2*) that were also confirmed in this study. The six novel SNPs in *PRPF6* were in high linkage disequilibrium and associated with *PRPF6* mRNA expression in lymphoblastoid cells from 373 Europeans in the 1000 Genomes Project. Taken together, our studies shed new light on the role of mRNA splicing genes in the development of lung cancer.

Lung cancer is a major challenge to human health and caused by multiple environment and genetic factors^1^. Smoking, radon gas, asbestos and air pollution have been established as the environment risk factors^2^, and genetic factors have also been clearly illustrated in familial^3^ and segregation studies^4^. Single nucleotide polymorphisms (SNPs) are the most common genetic variants in the human genome and have been shown to be associated with risk of human diseases, including cancers^5^. With a large sample size of study subjects, multiple-stage replication and high-throughput chips, genome-wide association studies (GWASs) have been shown to be a robust way of detecting genetic variants involved in susceptibility to cancer. To date, GWASs in different ethnic groups have successfully identified 30 loci in 12 chromosomal regions (3q28, 5p15.33, 6p21.1, 6p21.33, 6q22.1, 7p15.3, 10q25.2, 13q12.12,13q13.1, 15q25.1, 17q24.2, 22q12.2) that confer susceptibility to lung cancer^6^,^7^,^8^,^9^,^10^,^11^,^12^,^13^,^14^,^15^,^16^,^17^, among which, loci at 5p15.33, 6p21.33 and 15q25.1 were found to be risk factors for lung cancer in European descents.

Despite the great success achieved by GWASs, the identified SNPs by GWASs still only explain a small fraction of heritability of lung cancer, a phenomenon called “missing heritability”^18^. Therefore, many complementary approaches have been developed to improve the study power of GWASs. For example, one of such approaches is the pathway-based association analysis through additional imputation to increase the number of SNPs to be analyzed, which could detect the risk-associated genes with multiple, independent and small effect sizes. This approach could alleviate issues due to insufficient chip coverage of earlier GWASs, reduce the multiple-testing burden of GWAS, achieve consistent results across studies and provide understanding of genetic findings with some functional relevance^19^,^20^,^21^,^22^.

Among the biological candidate pathways for lung carcinogenesis, mRNA splicing, a modification of the pre-mRNA transcript in which introns are removed and exons are joined, is of significant importance^23^. In most of the circumstances, this pathway functions normally for the human genome to generate proteomic diversity sufficient for various biological processes. However, cancer cells may take advantage of this mechanism to produce aberrant proteins with added, deleted, or altered functional domains that contribute to carcinogenesis including lung cancer^24^. Recent studies had shown that somatic mutations and germline variants of some mRNA splicing-related genes were associated with development of lung cancer. For instance, Shen *et al*. found several SNPs in mRNA splicing associated genes (*SRSF7, PTBP2* and *HNRNPQ*) were associated with lung cancer risk in a Chinese population^25^. To date, however, only a limited number of candidate genes and SNPs in the pathway have ever been studied and reported. In the present study, we systematically selected 206 mRNA splicing-related genes and comprehensively investigated associations between 11,966 genetic variants of these genes and lung cancer risk using eight published lung-cancer GWAS datasets from the Transdisciplinary Research in Cancer of the Lung (TRICL) Consortium.

## Results

### Overall associations between SNPs in mRNA splicing-related genes and lung cancer risk in the TRICL Consortium

**Figure 1:**
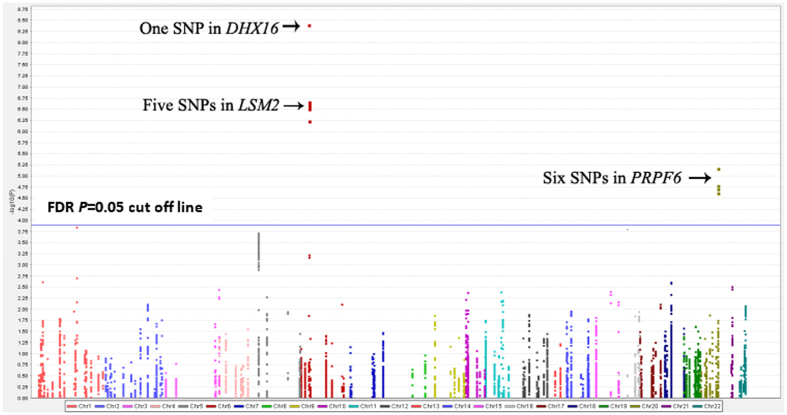
Association results of 11,966 SNPs in 206 mRNA splicing-related genes and lung cancer risk in the TRICL Consortium. SNPs are plotted on the X-axis according to their positions on each chromosome. The association *P* values with lung cancer risk are shown on the Y-axis (as −log10 *P* values). The 12 SNPs from three genes (*DHX16, LSM2* and *PRPF6*) were identified after the FDR correction.

**Figure 2:**
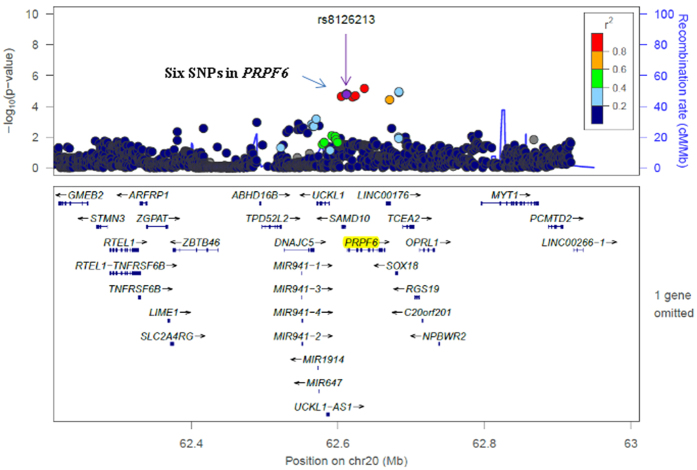
Regional plots and recombination rates of *PRPF6* (rs8126213 ± 500 kb) in the TRICL Consortium. *PRPF6* rs8126213 was shown in purple and the linkage disequilibrium (LD) values (r^2^) with the other SNPs are indicated by the heat scale. Other five *PRPF6* SNPs are shown in red color, which meant that they were in high LD with rs8126213 (r^2^ > 0.8).

Overall, 11,966 SNPs in 206 mRNA splicing-related genes in six GWAS datasets were selected from the TRICL Consortium (an imputated dataset that included 5,472,374 common SNPs), and their associations with lung cancer risk are shown in the Manhattan plot (Fig. 1). The sample size for each study had been listed in Table 1. After FDR correction (*P*_FDR corrected_ ≤ 0.05), the 12 SNPs in three genes remained statistically significant (Fig. 1). The most significant SNP for each gene was: rs75100087 in *PRPF6 (P* = 6.83E-06); rs115420460 in *DHX16 (P* = 3.93E-09) and rs114312980 in *LSM2 (P* = 2.19E-07). Their basic information and associations with lung cancer risk are listed in Table 2. SNPs in *DHX16* (rs115420460) and *LSM2* (rs114312980, rs115489726, rs115801685, rs114637560, rs115834633) were excluded from further analysis due to their locations within the previously identified lung cancer susceptible region Chr6p21.33 and in high LD with previously reported lung cancer GWAS SNPs. Specifically, as shown in Supplement Figure 2, rs115420460 in *DHX16* was in high LD (r^2^ > 0.6) with the previously reported lung cancer GWAS SNP rs4324798^9^. Similarly, Supplement Figure 3 shows that all of the five SNPs in *LSM2* were in high LD (r^2^ > 0.8) with SNP rs3117582 that was identified from a the previously published lung cancer GWAS^14^. Therefore, we focused on the remaining six newly identified SNPs in *PRPF6* (Table 2) for further analysis. Regional plot and recombination rates of *PRPF6* (rs8126213 ± 500 kb) in the TRICL consortium are presented in Fig. 2.

### LD analysis and SNP function annotation

The diagram of six *PRPF6* SNPs and their LD plot are shown in Supplement Figure 4b and 4c, respectively. High LD was observed between each pair of the six SNPs (r^2^ > 0.80), indicating that any of these six SNPs can be a good tag for others. Next, we exploited dbSNP function annotation (http://www.ncbi.nlm.nih.gov/projects/SNP/snp_ref.cgi?showRare=on&chooseRs=all&go=Go&locusId=24148) and another two online SNP function prediction tools: SNPfunc (http://snpinfo.niehs.nih.gov/snpinfo/snpfunc.htm) and regulomeDB (http://regulomedb.org/index) to assess their functionality (Supplementary Table 2).

### Expression quantitative Trait loci (eQTL) analysis

Expression quantitative trait loci (eQTL) analysis, which directly investigates the correlations between genetic variants and gene expression, has been gradually proved as an effect method to characterize the function of SNPs. In the present study, the eQTL analysis was performed by using the data available from lymphoblastoid cell lines derived from 373 individuals of European descendent (http://www.1000genomes.org/). Finally, all of these six SNPs in *PRPF6* were found to be significantly associated with expression levels of *PRPF6* in both additive and dominant models (Supplementary Figure 5 and Supplementary Table 2). We also queried the GTEx database (http://www.gtexportal.org/) and found that SNP rs8126213 was significantly correlated with mRNA expression levels of *PRPF6* in normal lung tissues (*P* = 1.50 × 10^−5^), which was consistent with the results in the lymphoblastoid cell lines. Similar results were found for other five SNPs (Supplementary Figure 6).

### Replication of *PRPF6* SNP (rs8126213) in another two lung-cancer GWASs

We then selected one of these six *PRPF6* SNPs (rs8126213) and investigated its association with lung cancer risk in two additional lung-cancer GWASs of Caucasian origin, Harvard Lung Cancer Study (984 cases and 970 controls) and Icelandic Lung Cancer Study (deCODE from the ILCCO) (1,319 cases and 26,380 controls). However, no significant association was observed in these two studies (*P* = 0.801 and 0.850, respectively), which may potentially result from a relatively limited sample size of the cases, although their effects were in the same direction as in the TRICL‐ILCCO studies, and the overall effect among all these eight GWASs remained significant (OR = 0.91, 95% CI = 0.87–0.95, *P* = 6.36E-05 in an additive genetic model) (Table 3). Similar effects were also observed on adenocarcinoma (OR = 0.91, 95% CI = 0.87–0.95, *P* = 0.002) and squamous cell carcinoma (OR = 0.90, 95% CI = 0.83–0.97, *P* = 0.005) in the subgroup analysis.

## Discussion

mRNA splicing is an important biological mechanism to regulate mRNA expression. Somatic mutations and germline variants of the mRNA splicing-related genes have been found to be associated with various kinds of cancers. For example, *SF3B1, U2AF1* and *SRSF2* mutations were observed in myeloid and lymphoid lineage tumors^26^. *SF3B1* mutations were commonly found in uveal melanomas^27^. Mutations affecting spliceosome genes that resulted in defective splicing were attributed to leukemia^28^. For lung cancer, it had been reported that *U2AF1* mutation was found in 3% of lung adenocarcinoma cases^29^. However, few studies reported a role of genetic variants in the etiology of cancer. For example, only one recent study found that genetic variants in three mRNA splicing-associated genes might modify individual susceptibility to lung cancer in a Chinese population^25^.

In the present study, we systematically evaluated the associations between 11,966 genetic variants in 206 mRNA splicing-related genes and lung cancer risk using the data from eight published GWAS datasets. To the best of our knowledge, this is the first and largest study focusing on exploring associations between SNPs from mRNA splicing-related genes and risk of lung cancer. Overall, our study identified six SNPs within mRNA splicing-related gene *PRPF6* that might play an important role in the development of lung cancer. Furthermore, all of these six SNPs were found to be associated with *PRPF6* mRNA expression in both lymphoblastoid cell lines and normal lung tissues of European descendent. Therefore, we proposed that these six SNPs were associated with abnormal expression of *PRPF6* and thus modified individual’s susceptibility to lung cancer. However, two major issues of the present study should be addressed. Firstly, the associations of these six SNPs with risk of lung cancer were observed in the six TRICL GWASs but not in Harvard and deCODE GWASs, probably due to a relatively limited sample size of the cases in the replication. Further replication studies are warranted to confirm our results. Secondly, the lung cancer-associated mRNA splicing-related genes identified in the previous Chinese study^25^ were not replicated in our study, which could be attributed to different genetic background or different environmental exposures as well as other unknown contributing factors between two populations.

*PRPF6* is located on chromosome 20q13.33 and is an essential member of the small nuclear ribonucleic proteins (snRNPs), playing an important role in mRNA splicing. For instance, a missense mutation in *PRPF6* impairs mRNA splicing and contributes to Autosomal-Dominant Retinitis Pigmentosa^30^. Its role in the development of cancer had also been reported in previous studies. For example, it was over-expressed in lung adenocarcinomas according to The Cancer Genome Atlas (TCGA) projects^31^. In addition, *PRPF6* was over-expressed in colon cancer^32^ to drive cancer proliferation by preferential splicing of genes associated with growth regulation^33^. Abnormal expression of *PRPF6* alters the constitutive and alternative splicing of a discrete number of genes, including an oncogenic isoform of the ZAK kinase^33^ that activates several cancer (including lung cancer)-related signaling pathways, such as those of NF-kB, Wnt/b catenin, AP1, ERK and JNK^34^,^35^. It should be mentioned that the protective alleles of these six SNPs were associated with both a decreased lung cancer risk and a decreased mRNA expression of *PRPF6*, which is consistent with the previous findings of overexpression of the gene in colon and lung cancers. Therefore, based on all these, we hypothesized of abnormal expression of *PRPF6* might contribute to development of lung cancer in the following way: the risk allele of *PRPF6* was associated with elevated *PRPF6* mRNA expression, then the oncogenic isoforms, such as the ZAK kinase, were generated as a result of abnormal splicing and activation in normal lung tissues, which finally resulted in lung carcinogenesis.

In summary, the present large-scale meta-analysis of eight published lung-cancer GWASs consisting of 14,463 lung cancer cases and 44,188 controls revealed a novel lung cancer susceptibility locus in the mRNA splicing-related gene *PRPF6* and provided some new insight into genetic architecture and carcinogenic mechanisms of lung cancer. Further validation and functional evaluation of this genetic variant are warranted to verify our findings.

## Materials and Methods

### Study populations

In the present study, we used the data from eight lung-cancer GWASs with a total of 14,463 lung cancer cases and 44,188 health controls. As shown in Table 1, the analysis included six GWAS datasets from the TRICL consortium (12,160 lung cancer cases and 16,838 controls of European ancestry) and another two datasets of lung cancer GWASs: the Caucasian origin-Harvard Lung Cancer Study (984 cases and 970 controls) and the Icelandic Lung Cancer Study (deCODE from the ILCCO) (1,319 cases and 26,380 controls). As described previously^16^, the TRICL six lung-cancer GWASs include the MD Anderson Cancer Center (MDACC) GWAS, the Institute of Cancer Research (ICR) GWAS, the National Cancer Institute (NCI) GWAS, the International Agency for Research on Cancer (IARC) GWAS, Lunenfeld-Tanenbaum Research Institute study (Toronto) GWAS and the German Lung Cancer Study (GLC) GWAS.

All of the subjects included in the analysis had provided a written informed consent. All methods were performed in accordance with the relevant guidelines and regulations, and all original studies were approved by the institutional review board from each of the participating institutions.

### GWAS genotyping, imputation and quality controls

GWASs included in the current study were performed by the following platforms: Illumina HumanHap 317, 317 + 240 S, 370 Duo, 550, 610 or 1 M arrays. Genotype imputation was conducted based on linkage disequilibrium (LD) information from the 1000 Genomes Project (phase I integrated release 3, March 2012) by IMPUTE2, MaCH or minimac software^16^. Imputed SNPs with information score <0.40 by IMPUTE2 or r^2^ < 0.30 by MaCH were excluded from further analysis. In addition, standard quality control on samples by excluding individuals with low call rate (<90%) and extremely high or low heterozygosity (*P* < 1.0 × 10^−4^), as well as those estimated to be of non-European ancestry (using the HapMap phase II CEU, JPT/CHB and YRI populations as reference) were conducted among all of the studies.

### Genes and SNPs selection

The mRNA splicing-related genes were selected with the combination of two online databases commonly exploited in the gene-set enrichment analysis: Molecular Signatures Database (http://www.broadinstitute.org/gsea/index.jsp) and Genecards (http://www.genecards.org/). Overall, 206 mRNA splicing-related genes were selected (Supplementary Table 1), which included a total of 11,966 genotyped or imputed common SNPs extracted from these genes (include 2-kb upstream and downstream of genes) among the TRICL Consortium with the following inclusion criteria: 1) Genotyping call rate ≥ 90%; 2) Minor allele frequency (MAF) ≥ 5% among Europeans; and 3) Hardy Weinberg Equilibrium exact *P* value ≥ 10^−5^. The detailed work-flow can be found in Supplementary Figure 1.

### Statistical analysis

The associations between SNPs and lung cancer risk were evaluated using an additive genetic model by R (v2.6), Stata (v10, State College, Texas, US) and PLINK (v1.06) software^36^. For the studies of ICR, MDACC, IARC, Toronto, NCI and Harvard, top significant principle components were included in the analysis to control for population stratification that might cause inflation of test statistics, while for the deCODE study, genomic control method was used to adjusted for population stratification. No population structure was found in the German Lung Cancer Study (GLC). With the application of the inverse variance method, a meta-analysis under the fixed and random-effects models was performed on the results of a log-additive model for 11,966 SNPs. In order to assess the heterogeneity among studies, the Cochran’s Q statistic to test for heterogeneity and the *I*^2^ statistic to quantify the proportion of the total variation due to the heterogeneity were calculated^31^. A fixed-effects model was applied, if no heterogeneity existed among studies (*P*_Q-test_ > 0.10 and *I*^2^ < 25%); otherwise, a random-effects model was chosen. The Benjamini–Hochberg false discovery rate (FDR) procedure was employed for the correction of multiple testing with a cutoff value ≤ 0.05.

Regional association plots were generated with LocusZoom on the basis of 1000 Genomes European (EUR) reference data (phase I integrated release 3, March 2012)^37^. Haploview v4.2 was employed to construct the Manhattan and LD plot. All analyses were conducted with SAS (version 9.1.3; SAS Institute, Cary, NC, USA) unless specified otherwise.

### *In silico* SNP function annotation

To prioritize functional SNPs, three *in silico* tools: dbSNP func annotation (http://www.ncbi.nlm.nih.gov/projects/SNP/), SNPinfo (http://snpinfo.niehs.nih.gov/), and RegulomeDB (http://regulomedb.org/) were employed. Furthermore, the associations between SNPs and *PRPF6* mRNA expression were performed using lymphoblastoid cell expression data from 1000 Genomes Project European populations (EUR, 373 individuals) (phase I integrated release 3, March 2012)^38^ by linear regression model.

## Additional Information

**How to cite this article:** Pan, Y. *et al*. Associations between genetic variants in mRNA splicing-related genes and risk of lung cancer: a pathway-based analysis from published GWASs. *Sci. Rep.* **7**, 44634; doi: 10.1038/srep44634 (2017).

**Publisher's note:** Springer Nature remains neutral with regard to jurisdictional claims in published maps and institutional affiliations.

## Supplementary Material

Supplementary Tables and Figures

## Figures and Tables

**Table 1:** Characteristic information of the study populations.

Study	Controls	Lung cancer patients		
		All	AD^10^	SQ^11^
ICR^1^	5200	1952	465	611
MDACC^2^	1134	1150	619	306
IARC^3^	3791	2533	517	911
NCI^4^	5736	5713	1841	1447
Toronto^5^	499	331	90	50
GLC^6^	478	481	186	97
**TRICL**^7^	16838	12160	3718	3422
Harvard^8^	970	984	597	216
deCODE^9^	26380	1319	547	259
**All combined**	44188	14463	4862	3897

^1^ICR: the Institute of Cancer Research Genome-wide Association Study, UK;

^2^MDACC: the MD Anderson Cancer Center Genome-wide Association Study, US;

^3^IARC: the International Agency for Research on Cancer Genome-wide Association Study, France;

^4^NCI: the National Cancer Institute Genome-wide Association Study, US;

^5^Toronto: the Lunenfeld-Tanenbaum Research Institute Genome-wide Association Study, Toronto, Canada;

^6^GLC: German Lung Cancer Study, Germany;

^7^TRICL: GWASs datasets combined by six GWASs of ICR, MDACC, IARC, NCI, Toronto and GLC;

^8^Harvard: Harvard Lung Cancer Study, US;

^9^deCODE: Icelandic Lung Cancer Study, Iceland;

^10^AD: adenocarcinoma;

^11^SQ: squamous cell carcinoma.

**Table 2:** mRNA splicing-related genes SNPs and lung cancer risk in the TRICL^1^ Consortium with FDR corrected *P* ≤ 0.05.

ID	SNP	Chr: Position^2^	GENE	Allele^3^	EAF^4^	Q^5^	*I*^2^ 5	Effects^6^	OR (95% CI)^7^	*P*^7^	FDR^8^
1	rs115420460	6: 30618906	*DHX16*	A/G	0.11	0.67	0.00	++++++	1.18 (1.12–1.25)	3.93E-09	<0.0001
2	rs114312980	6: 31768799	*LSM2*	A/C	0.11	0.23	26.77	++++++	1.20 (1.12–1.29)	2.19E-07	0.0007
3	rs115489726	6: 31766660	*LSM2*	C/T	0.11	0.24	25.69	++++++	1.20 (1.12–1.29)	2.54E-07	0.0007
4	rs115801685	6: 31772093	*LSM2*	C/A	0.11	0.23	27.36	++++++	1.20 (1.12–1.29)	2.61E-07	0.0007
5	rs114637560	6: 31765864	*LSM2*	T/A	0.15	0.27	22.42	+−++++	1.14 (1.07–1.21)	3.04E-07	0.0007
6	rs115834633	6: 31765984	*LSM2*	G/A	0.11	0.20	30.79	++++++	1.20 (1.12–1.30)	5.75E-07	0.0011
7	rs116165844	20: 62610556	*PRPF6*	G/T	0.13	0.89	0.00	−−−−−−	0.89 (0.85–0.94)	1.62E-05	0.0197
8	rs8126213	20: 62611478	*PRPF6*	G/A	0.13	0.89	0.00	−−−−−−	0.89 (0.85–0.94)	1.64E-05	0.0197
9	rs147176547	20: 62613001	*PRPF6*	C/G	0.14	0.98	0.00	−−−−−−	0.90 (0.85–0.94)	1.65E-05	0.0197
10	rs112219537	20: 62620029	*PRPF6*	G/A	0.13	0.96	0.00	−−−−−−	0.90 (0.85–0.94)	2.37E-05	0.0237
11	rs113450630	20: 62623703	*PRPF6*	C/T	0.14	0.98	0.00	−−−−−−	0.90 (0.85–0.94)	1.93E-05	0.021
12	rs75100087	20: 62636139	*PRPF6*	C/T	0.14	0.97	0.00	−−−−−−	0.89 (0.85–0.94)	6.83E-06	0.0117

^1^TRICL: GWASs datasets combined by six GWASs of ICR, MDACC, IARC, NCI, Toronto and GLC.

^2^Based on NCBI build 37 of the human genome.

^3^Reference allele/effect allele.

^4^EAF, effect allele frequency.

^5^Fixed-effects models were used when no heterogeneity was found between studies (Q > 0.10 and *I*^2^ < 25.0); otherwise, random-effects models were used.

^6^+ means positive association, and − means negative association.

^7^Meta-analysis of additive results from six lung cancer GWASs.

^8^FDR, false discovery rate.

**Table 3:** Summary association results of *PRPF6* rs8126213 (G > A) in all of the eight lung cancer GWASs.

Study population	Sample size		Overall (N = 14463)		AD (N = 4862)		SQ (N = 3897)	
	Cases	Controls	OR (95% CI)^*^	P^*^	OR (95% CI)^*^	P^*^	OR (95% CI)^*^	P^*^
**TRICL**	12160	16838	0.89 (0.85–0.94)	1.65E-05	0.88 (0.81–0.95)	9.0E-04	0.88 (0.82–0.96)	2.20E-03
ICR	1952	5200	0.94 (0.85–1.05)	0.303	0.94 (0.77–1.15)	0.572	0.92 (0.77–1.20)	0.374
MDACC	1150	1134	0.87 (0.73–1.04)	0.120	0.87 (0.71–1.07)	0.199	0.84 (0.64–1.11)	0.219
IARC	2533	3791	0.91 (0.81–1.01)	0.075	0.93 (0.76–1.13)	0.456	0.91 (0.78–1.06)	0.238
NCI	5713	5736	0.87 (0.81–0.95)	8.36E-04	0.86 (0.77–0.97)	0.012	0.85 (0.75–0.96)	7.92E-03
Toronto	331	499	0.86 (0.62–1.20)	0.379	0.77 (0.47–1.27)	0.314	0.97 (0.51–1.83)	0.923
GLC	481	478	0.83 (0.63–1.10)	0.195	0.67 (0.45–0.98)	0.041	1.01 (0.64–1.58)	0.979
**Harvard and deCODE**	2303	27350	0.98 (0.89–1.09)	0.772	0.97 (0.84–1.12)	0.683	1.02 (0.82–1.26)	0.875
Harvard	984	970	0.97 (0.79–1.20)	0.801	0.94 (0.74–1.18)	0.576	0.97 (0.68–1.40)	0.886
deCODE	1319	26380	0.99 (0.87–1.12)	0.850	0.99 (0.82–1.20)	0.940	1.04 (0.80–1.37)	0.761
**All combined**	14463	44188	0.91 (0.87–0.95)	6.36E-05	0.91 (0.87–0.95)	1.70E-03	0.90 (0.83–0.97)	4.80E-03

Abbreviations: TRICL: GWASs datasets combined by six GWASs of ICR, MDACC, IARC, NCI, Toronto and GLC; ICR: the Institute of Cancer Research Genome-wide Association Study, UK; MDACC: the MD Anderson Cancer Center Genome-wide Association Study, US; IARC: the International Agency for Research on Cancer Genome-wide Association Study, France; NCI: the National Cancer Institute Genome-wide Association Study, US; Toronto: the Lunenfeld-Tanenbaum Research Institute Genome-wide Association Study, Toronto, Canada; GLC: German Lung Cancer Study, Germany; Harvard: Harvard Lung Cancer Study, US; deCODE: Icelandic Lung Cancer Study, Iceland; AD: adenocarcinoma; SQ: squamous cell carcinoma; ^*^Meta-analysis of results from additive model.
