# Supplementary material for: Associations between genetic variants in mRNA splicing-related genes and risk of lung cancer: a pathway-based analysis from published GWASs
Source: Sci Rep. 2017 Mar 17;7:44634. doi: 10.1038/srep44634 (PMC5356340; doi:10.1038/srep44634)
Supplement: Supplementary Tables and Figures [file srep44634-s1.doc]

**Associations between genetic variants in mRNA splicing-related genes and risk of lung cancer: a pathway based analysis from published GWASs**

Yongchu Pan^1,2,3*^, Hongliang Liu^1,3*^, Yanru Wang^1,3^, Xiaozheng Kang^1,3^, Zhensheng Liu^1,3^, Kouros Owzar^1,4^, Younghun Han^5^, Li Su^6,7^, Yongyue Wei^6,7^, Rayjean J. Hung^8^, Yonathan Brhane^8^, John McLaughlin^9^, Paul Brennan^10^, Heike Bickeböller^11^, Albert Rosenberger^11^, Richard S. Houlston^12^, Neil Caporaso^13^, Maria Teresa Landi^13^, Joachim Heinrich^14^, Angela Risch^15^, Xifeng Wu^16^, Yuanqing Ye^16^, David C. Christiani^6,7^, Christopher I. Amos^5^, and Qingyi Wei^1,3**^.

**Supplementary Table 1.** Selection of mRNA splicing-related genes in MSigDB and Genecards databases

| Database |  | | Name of Pathway | Genes |
| --- | --- | --- | --- | --- |
| **MSigDB** |  | |  |  |
| Reactome |  | | mRNA Splicing | 112 |
|  |  | | mRNA Splicing-minor pathway | 45 |
| **Genecards** |  | | mRNA Splicing | 201 |
| **Combined** |  | | mRNA Splicing genes | 206 |
|  | |  | | |

**Supplementary Table 2. Function annotation and prediction of six SNPs of *PRPF6 in silico***

| SNPs | Allele | dbSNP func annotation | SNPinfo | RegulomeDB Score | Association with *PRPF6* expression (*P*_add_ / *P*_dom_) |
| --- | --- | --- | --- | --- | --- |
| rs116165844 | G/T | 5’ near gene | - | 2b: TF binding+any motif+DNase Footprint +DNase peak | 0.042/0.039 |
| rs8126213 | G/A | 5’ near gene | TFBS* | 4: TF binding+DNase peak | 0.042/0.039 |
| rs147176547 | C/G | Intron | - | 2b: TF binding+any motif+DNase Footprint+DNase peak | 0.028/0.025 |
| rs112219537 | G/A | Intron | - | - | 0.029/0.026 |
| rs113450630 | C/T | Intron | - | - | 0.028/0.025 |
| rs75100087 | C/T | Intron | - | 5: TF binding or DNase peak | 0.028/0.025 |

*TFBS: Transcription factor binding site

＃expression quantitative loci analyses from 373 individuals of European descendent (http://www.1000genomes.org/)

**206 mRNA splicing-related genes**

**MisgDB: 112 genes**

**Genecards: 201 genes**

**Combined: 206 genes**

**2 kb upstream and downstream**

**Genotyping call rate≥90%**

**Minor allele frequency ≥ 5%**

**HWE *P* ≥10^-5^**

**11,966 SNPs**

**Six SNPs among *PRPF6***

**Meta-analysis**

**among TRICL Consortium**

**12,160 cases and 16,838 controls**

**Additive genetic model**

**12 SNPs with FDR *P*≤0.05**

**Exclude: SNPs in *DHX16* and *LSM2*-In high LD with previously reported lung cancer GWAS SNPs**

**Include: 6 SNPs in *PRPF6* (novel)**

**LD analysis**

**SNP function prediction**

**eQTL analysis**

**Replication on one tagSNP rs8126213**

**Replication and overall analysis with Harvard and deCODE lung cancer GWASs**

**Supplementary Figure 1.** Flow-chart of the present study. HWE: Hardy Weinberg Equilibrium; FDR: false discovery rate; LD: Linkage disequilibrium; eQTL: expression quantitative loci

**Supplementary Figure 2.** rs115420460 (new SNP ID: rs9262135) in *DHX16* identified in the TRICL Consortium was in moderate to high linkage disequilibrium (LD) with those previously reported lung cancer GWAS SNPs (*P* < 10^-8^)

**Supplementary Figure 3.** Five SNPs in *LSM2* identified in the TRICL Consortium were in moderate to high linkage disequilibrium (LD) with previously reported lung cancer GWAS SNPs (*P* < 10^-8^).

**
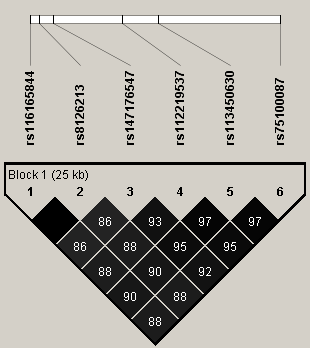
**

Exon

**rs116165844**

**rs8126213**

**rs147176547**

**rs112219537**

**rs113450630**

**rs75100087**

UTR

***PRPF6***

20p13

P12.3

20p12.1

20q12

13.12

q13.2

q13.33

**Chr20**

**A**

**B**

**C**

rs8126213

tagSNP

**Supplementary Figure 4.** (A) Chromosome position of *PRPF6*; (B) Diagram of six *PRPF6* SNPs; (C) linkage disequilibrium (LD) values (r^2^) among these six *PRPF6*. High LD was observed between each of the two SNPs. Among them, rs8126213 was selected as tagSNP due to its potential functionality predicted by dbSNP func annotation, SNPfunc.


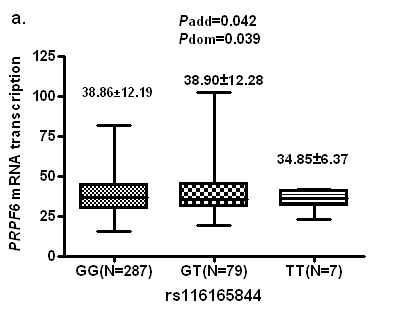

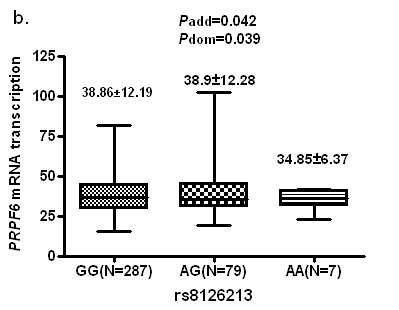

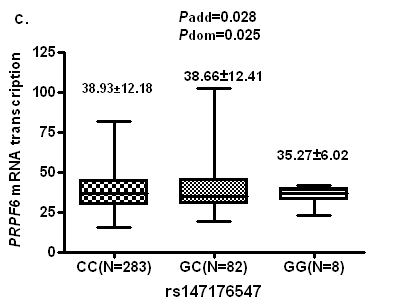


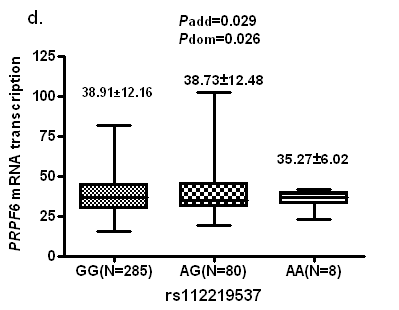

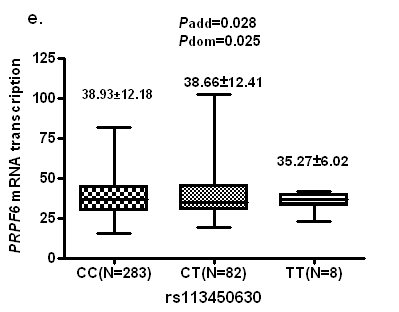

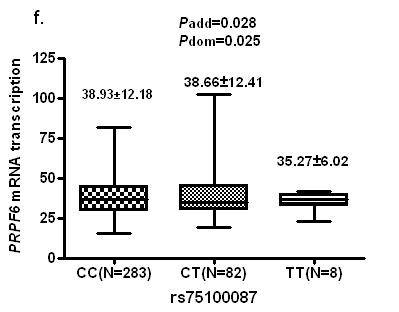


**Supplementary Figure 5.** Association between the six *PRPF6* SNPs and *PRPF6* mRNA expression. All the data were from 373 Europeans of 1000 Genomes Project. The association between genotypes and mRNA expression levels was evaluated using a linear regression model. Associations were calculated with additive and dominant genetic model.


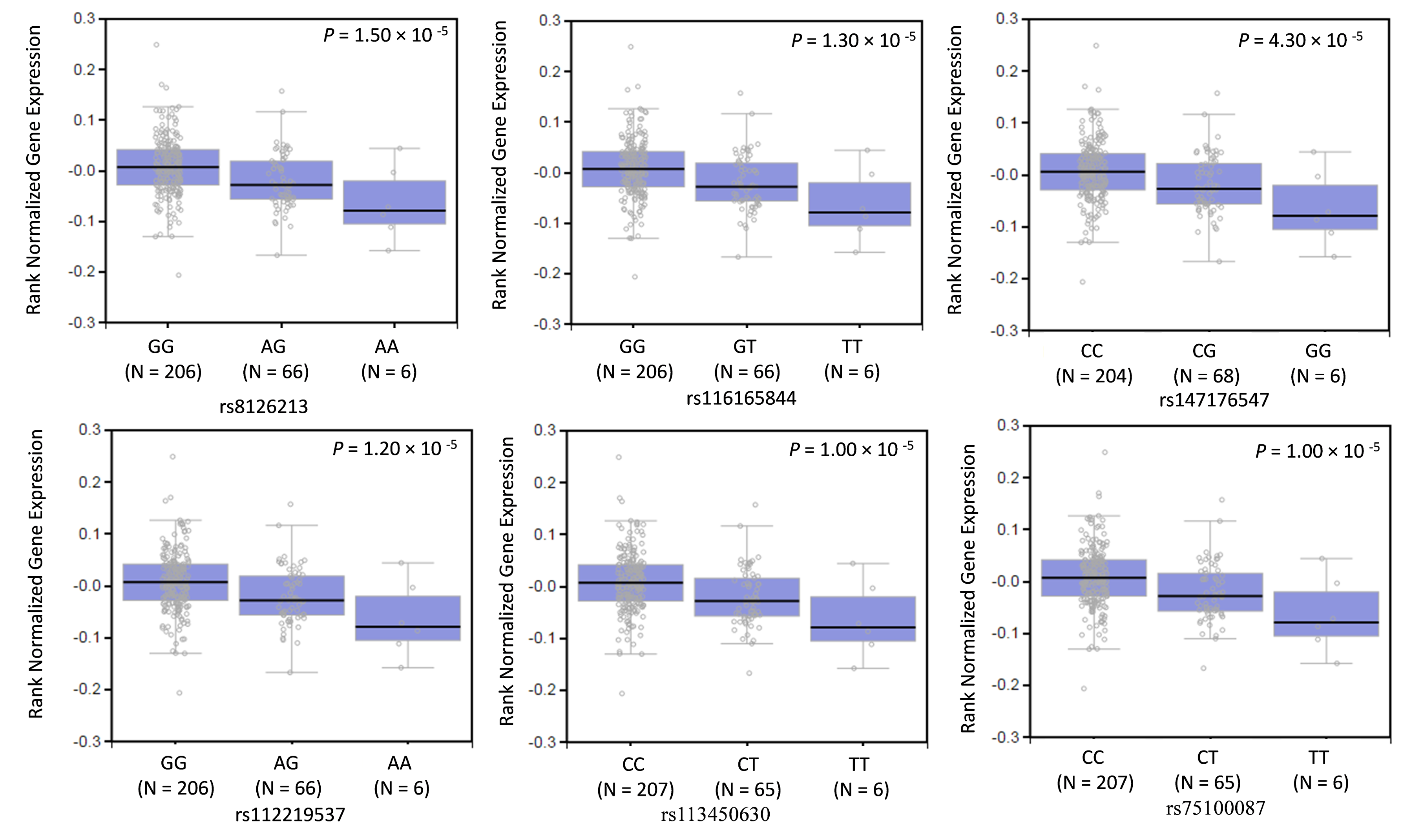


**Supplementary Figure 6.** Association between the six *PRPF6* SNPs and *PRPF6* mRNA expression in normal lung tissues. All plots were generated from the Genotype-Tissue Expression (GTEx) project (http://www.gtexportal.org/home/).
